# Abatement of the Stimulatory Effect of Copper Nanoparticles Supported on Titania on Ovarian Cell Functions by Some Plants and Phytochemicals

**DOI:** 10.3390/nano10091859

**Published:** 2020-09-17

**Authors:** Alexander V. Sirotkin, Monika Radosová, Adam Tarko, Zuzana Fabova, Iris Martín-García, Francisco Alonso

**Affiliations:** 1 Department of Zoology and Anthropology, Constantine the Philosopher University, Tr. A Hlinku 1, 949 74 Nitra, Slovakia; monika.radosova@gmail.com (M.R.); tarko.adam.000@gmail.com (A.T.); zuzka.fabova@gmail.com (Z.F.); 2 Instituto de Síntesis Orgánica (ISO) and Departamento de Química Orgánica, Facultad de Ciencias, Universidad de Alicante, Apdo., 99, 03080 Alicante, Spain; irismartingarcia@gmail.com

**Keywords:** apoptosis, copper nanoparticles, hormones, buckwheat, vitex, rutin, apigenin, ovary, phytochemicals, proliferation

## Abstract

The application of nanoparticles has experienced a vertiginous growth, but their interaction with food and medicinal plants in organisms, especially in the control of reproduction, remains unresolved. We examined the influence of copper nanoparticles supported on titania (CuNPs/TiO_2_), plant extracts (buckwheat (*Fagopyrum esculentum*) and vitex (*Vitex agnus-castus*)), phytochemicals (rutin and apigenin), and their combination with CuNPs/TiO_2_ on ovarian cell functions, using cultured porcine ovarian granulosa cells. Cell viability, proliferation (PCNA accumulation), apoptosis (accumulation of bax), and hormones release (progesterone, testosterone, and 17*β*-estradiol) were analyzed by the Trypan blue test, quantitative immunocytochemistry, and ELISA, respectively. CuNPs/TiO_2_ increased cell viability, proliferation, apoptosis, and testosterone but not progesterone release, and reduced the 17*β*-estradiol output. Plant extracts and components have similar stimulatory action on ovarian cell functions as CuNPs/TiO_2_, but abated the majority of the CuNPs/TiO_2_ effects. This study concludes that (1) CuNPs/TiO_2_ can directly stimulate ovarian cell functions, promoting ovarian cell proliferation, apoptosis, turnover, viability, and steroid hormones release; (2) the plants buckwheat and vitex, as well as rutin and apigenin, can promote some of these ovarian functions too; and (3) these plant additives mitigate the CuNPs/TiO_2_’s activity, something that must be considered when applied together.

## 1. Introduction

Nanotechnology and the applications of metal nanoparticles (i.e., particles with a diameter < 100 nm) have rapidly developed in recent years [1,2,3,4]. In particular, nanoparticles of the relatively cheap and abundant copper (CuNPs) have found multiple applications in diverse areas, including catalysis [5] and materials science [6,7]. However, CuNPs have also been exploited for disinfection purposes because of their effective antimicrobial properties, in medicinal chemistry or as farm animal food additives [8,9,10]. Nevertheless, copper nanoparticles can adversely influence various biological processes [11,12,13] and produce some toxicological effects on individual organisms [14].

Assays conducted on rodents proved the ability of both bulk copper and CuNPs to diminish the levels of blood gonadal and gonadotropin steroid hormones to cause degenerative alterations in gonads, ovarian follicular atresia, and to inhibit gamete and embryogenesis [15,16,17]. This detrimental behavior could be attributed to the ease of CuNPs to suppress the function of some antioxidative enzymes and to produce ovarian cell apoptosis [16]. Other surveys, however, did not report any unfavorable effect of CuNPs on various ovarian follicles of mice [15] and pregnancy [17]. Some studies revealed that copper can have an important role in stimulating porcine pituitary gonadotropin secretion [15], on the ovulation rate [18], release of insulin-like growth factor I and progesterone, and on the suppressed apoptosis and proliferation in cultured granulosa cells [15,19]. Recent in vitro studies demonstrated both positive and adverse effects of various unsupported and supported CuNPs on cell viability, proliferation, apoptosis, and steroid hormones release in porcine ovarian granulosa cells [20]. Therefore, it is desirable to synthesize proper CuNPs that can be applied with minimal toxic effects. Moreover, some CuNPs with stimulatory influence on reproductive processes could be useful as novel biostimulators of reproduction and, therefore, in animal production, biotechnology, and assisted reproduction.

We have been involved in the development of methods to prepare metal nanoparticles [21], copper nanoparticles among them [22], which have found application as chemical catalysts either unsupported [23] or supported on a variety of inorganic supports, including zeolite Y [24], charcoal [25], and titania [26]. A previous comparison of CuNPs of different size, morphology, and inorganic support enabled us [20] to identify CuNPs supported on titania nanopowder (CuNPs/TiO_2_) as a nanoparticle combination with a potent direct effect on ovarian cells.

On the other hand, medicinal plants have been used in traditional medicine for centuries. That is why there is a general upsurge of interest in the application of plant extracts to prevent or treat manifold diseases [27,28,29,30]. It is known that some medicinal plants or their isolated chemical components (i.e., phytochemicals) are able to affect reproduction. They can be administered instead or in addition to classical pharmacological drugs [31,32,33], though some interference between the plant and the pharmacological preparation in their effect on female reproduction is possible [34].

In this context, buckwheat (*Fagopyrum esculentum*) is a medicinal and food plant containing a large variety of polyphenolic antioxidants (including rutin and quercetin) with numerous health benefits [35], the action of which on reproductive processes remains, however, unknown. Vitex (*Vitex agnus-castus*) is another potential regulator of reproduction and health. The presence of antioxidants and phytoestrogens in vitex makes it useful for the treatment of polycystic ovarian syndrome and the restoration of sex steroid release and estrous cycle [36,37,38], for the therapy of premenstrual and postmenstrual syndromes [39,40] and as a remedy for infertility [39]. Due to its ability to promote apoptosis, it can be also beneficial for the prevention and cure of cancer [41,42].

The main biological effects of medicinal and food plants are related to the presence of polyphenols with antioxidant and phytoestrogen activity. One of these polyphenols is the flavonoid rutin, which is able to promote bone cell proliferation [43]. It can ameliorate testicular cell apoptosis, damage of spermatogenesis, and the reduction of spermatogenesis induced by ischemia-reperfusion injury [44] and restraint [45] and oxidative [46] stress. Rutin was able to prevent also the symptoms of polycystic ovarian syndrome [47]. Another flavonoid-type phytoestrogen, apigenin, is able to suppress proliferation and viability, and to promote oxidative stress and apoptosis in non-ovarian [48,49] and ovarian [50] cancer cells. It can restore ovarian folliculogenesis, ovulation, and steroidogenesis during polycystic ovarian syndrome [51]. Furthermore, apigenin was able to prevent ovarian damage induced by ischemia-reperfusion injury [52].

The available literature demonstrates the therapeutic potential of the aforementioned plants and phytochemicals in the treatment of some reproductive disorders. However, there is no available information concerning their action, for instance, by their routinely daily intake, on healthy reproductive systems. Furthermore, CuNPs, medicinal/food plants and phytochemicals can be applied to regulate reproduction and reproductive disorders, but their functional interrelationships within the ovary by their joint application have not been elucidated yet.

Our present study is aimed to understand whether basic ovarian cell functions can or cannot be directly affected by: (a) CuNPs/TiO_2_, (b) some medicinal/food plants (buckwheat and vitex) and/or their polyphenol constituents (rutin and apigenin), and (c) the combination of CuNPs/TiO_2_ and the plant/phytochemical. For this purpose, we compared the influence of CuNPs/TiO_2_ (0, 1, 10, or 100 µg/mL), buckwheat, vitex, rutin, and apigenin (10 µg/mL) when given individually and when CuNPs/TiO_2_ was combined with plant additives. We compared the activity of these treatments on cultured porcine ovarian granulosa cells by studying cell viability, the accumulation of bax (a cytoplasmic apoptosis marker) [53,54], PCNA (a proliferation marker) [55], and the release of the steroid hormones testosterone, progesterone, and 17*β*-estradiol, the regulators and markers of ovarian cell functions [56]. This survey reveals that CuNPs/TiO_2_ can boost some of the aforementioned ovarian cell functions, as buckwheat, vitex, rutin, and apigenin also do. However, the activity CuNPs/TiO_2_ is significantly depleted in the presence of these plant additives.

## 2. Materials and Methods

### 2.1. Preparation of Copper Nanoparticles Supported on Titania (CuNPs/TiO_2_)

The methodology of Alonso’s group [21] was applied for the synthesis of CuNPs/TiO_2_. In this protocol, a suspension was prepared in a Schlenk flask containing lithium powder (14 mg, 2.0 mmol, Medalchemy S.L.) and 4,4′-di-*tert*-butylbiphenyl (27 mg, 0.1 mmol; DTBB, Sigma-Aldrich, St. Louis, MO, USA) in dry tetrahydrofuran as a solvent (THF, 20 mL) under argon at room temperature. Anhydrous CuCl_2_ (134 mg, 1.0 mmol; 97%, Sigma-Aldrich, St. Louis, MO, USA) was added to this suspension. The initially formed dark-green reaction mixture changed to black, as a result of the formation of CuNPs. Then, additional dry THF (18 mL) and TiO_2_ (1.28 g, titania anatase nanopowder, Alfa Aesar, Ward Hill, MA, USA) were added to the above suspension. The reaction mixture was stirred at room temperature for 1 h, followed by filtration and drying under air.

### 2.2. Isolation and Culture of Granulosa Cells

The granulosa cells were isolated from the ovaries of non-cycling pubertal gilts (180 days age, approximately), and were processed and cultured as previously described [57,58,59,60,61]. Briefly, the collected granulosa cells (at 10^6^ cells/mL concentration) were precultured in sterile Dulbecco’s modified Eagle medium (DMEM)/F12 1:1, containing 10% fetal calf serum (both from BioWhittakerTM, Verviers, Belgium) and 1% antibiotic-antimycotic solution (Sigma-Aldrich, St. Louis, MO, USA) in 16-well (200 µL/well) chamber slides (Nunc Inc., International, Naperville, IL, USA) during 3–4 days. Buckwheat (*Fagopyrum esculentum*), vitex (*Vitex agnus-castus*; both from F-DENTAL Hodonín s.r.o., Hodonín, Czech Republic), rutin (WallMark, a.s., Třinec, Czech Republic), and apigenin (Merck KGaA, Darmstadt, Germany) were commercially available. The plants were minced for 2 min in a coffee mill. Thereafter, either the plant powder or the plant components apigenin and rutin were dissolved in 50 µL of DMSO in order to make stock solutions of 1 mg/mL. These stock solutions were dissolved in the culture medium immediately before their addition to the cells, in such a way that the final concentration of DMSO did not exceed 0.001%. Previous studies revealed no substantial effects of 0.001% DMSO on ovarian cell function and viability. Controls included ovarian cells cultured in the incubation medium (with 0.001% DMSO) without treatment and the medium incubated without cells (blank control).

The original medium was replaced with one of the same composition without and with CuNPs/TiO_2_, at the concentrations of 0, 1, 10, or 100 µg/mL, with buckwheat, vitex, rutin, and apigenin, each at 10 µg/mL. In addition, CuNPs/TiO_2_ (0, 1, 10, or 100 µg/mL) was combined with each of the plant additives listed above (10 µg/mL). In this case, the plant additive was first added to the cell culture and, after 10–15 min, CuNPs/TiO_2_ was added to the resulting medium. These doses correspond to those of CuNPs that can be ingested and that have been applied in previous animal experiments, both in vivo and in vitro [20,58,59,60,61]. The dose of the plant additives was selected on the basis of the doses used in the corresponding previous in vitro experiments [41,43,45,49,50]. These substances were dissolved in the culture medium just before their addition to the cells. The cell and the incubation medium were analyzed after two days of culture.

### 2.3. Cell Viability Test

The Trypan blue exclusion test was used to evaluate the cell viability, according to Strober [62]. Succinctly, removal of the medium from the culture plates was done after incubating the granulosa cells. Then, the Trypan blue staining (Sigma-Aldrich, Hamburg, Germany) was applied to the cell monolayer for 15 min. After removal of this dye, the plates were subjected to washing (twice with the physiological solution) and to microscopic inspection (magnification: 400×). The ratio of dead (stained) cells to the total cell count was calculated.

### 2.4. Immunocytochemical Analysis of Proliferation and Apoptosis Markers

**Figure 1:**
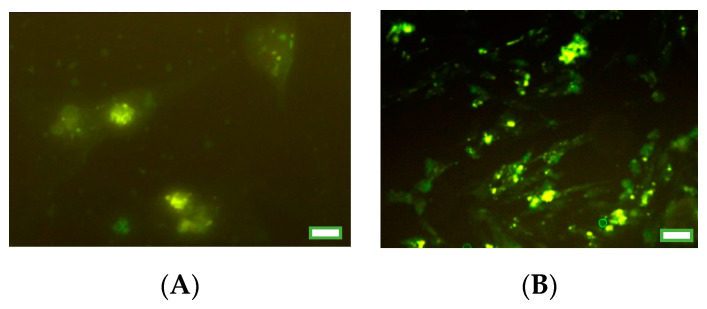
Fluorescence microscopy images of porcine granulosa cells containing fluorescein isothiocyanate (FITC, green fluorescence), indicating the proliferating cell nuclear antigen (PCNA; **A**) or Bcl-2-associated X protein (bax; **B**). Scale bars represent 10 µm.

Immunocytochemistry was used to detect the presence of PCNA and bax in the cells, as described previously [19,20,57,58,59,60,61], by means of primary monoclonal antibodies against PCNA and bax (dilution 1:500; from Santa Cruz Biotechnology, Inc., Santa Cruz, CA, USA), secondary swine antibodies against mouse IgG labeled with horseradish peroxidase (dilution 1:1000; Servac, Prague, Czech Republic) and visualized by DAB-substrate staining (Roche Diagnostics GmbH, Manheim, Germany). In some cases, the assay was validated by these primary antibodies and secondary polyclonal goat antibodies against mouse IgGs, labeled with fluorescein isothiocyanate (FITC, dilution 1:1000; Santa Cruz Biotechnology, Inc., Santa Cruz, CA, USA; Figure 1). The negative controls were performed with cells processed without the primary or secondary antibody. The cells were inspected with the aid of a light and a fluorescence microscope (Leica GmbH, Wetzlar, Germany). The cells showing a signal larger than that of the levels of the background negative controls were considered positive. The percentage of cells containing a visible signal–marker of PCNA and bax was calculated relative to the total cell number.

### 2.5. Immunoassay of Hormones

Progesterone, testosterone, and 17*β*-estradiol concentrations were determined from 25 µL aliquots of the incubation medium through the enzyme-linked immunosorbent assay (ELISA), utilizing ELISA’s kits (LDN Immunoassays and Services, Nodhorn, Germany), following the instructions of the manufacturer. The details of the assay were reported previously [20]. Validation of all the assays was accomplished by dilution tests of culture medium samples.

### 2.6. Statistical Analysis

Each experiment was repeated thrice using ovaries from different animals (10–15 ovaries per experiment). In some experiments, all the treatments were tested at once, in other experiments the effects of only part of the additives were investigated (for example, CuNPs/TiO_2_ alone and in combination with rutin or apigenin). The presented data are the summarized results obtained from, at least, three independent experiments. Each group of experiments was represented by four culture wells. Immunocytochemical and viability tests were performed on, at least, 1000 cells per group. By using ELISA, blank control values were subtracted from those determined in the cell-conditioned medium in order to exclude any non-specific background (<13% of the total values). Secretion rates were calculated for 10^6^ cells/day. Differences between groups were evaluated using the Shapiro–Wilk’s normality and Student’s *t*-tests and Sigma Plot 11.0 (Systat Software, GmbH, Erkhart, Germany). The data was expressed as the mean ± standard error of mean (SEM). Differences were considered statistically significant at *p* ≤ 0.05.

## 3. Results

**Figure 2:**
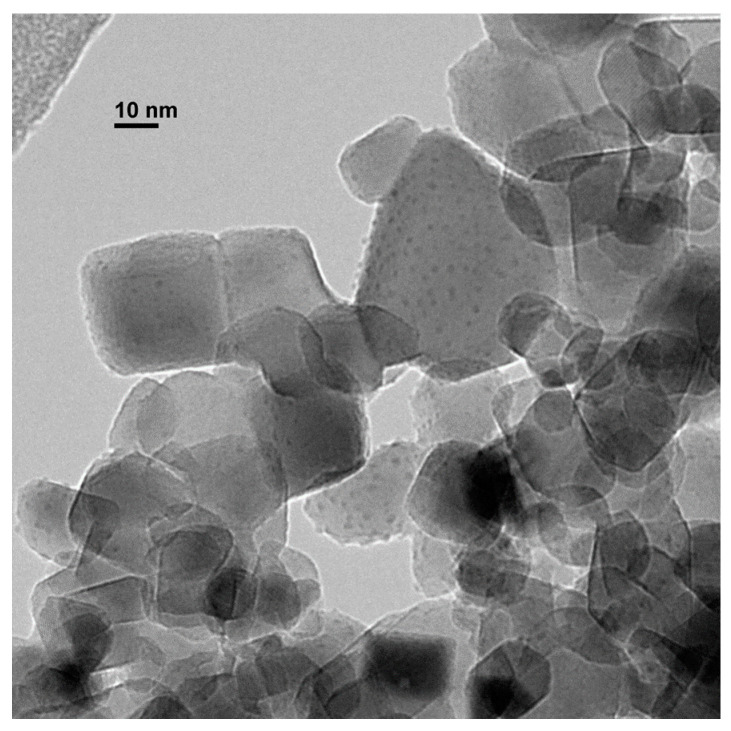
Transmission electron microscopy micrograph of CuNPs/TiO_2_.

The solid composed of copper nanoparticles on titania (CuNPs/TiO_2_) was characterized by transmission electron microscopy (TEM), showing well dispersed spherical CuNPs of an 0.98 ± 0.42 nm average size (Figure 2). The copper loading was determined to be 1.9 wt% by inductively coupled plasma optical emission spectroscopy (ICP-OES). The oxidation state of the copper species was analyzed by X-ray photoelectron spectroscopy (XPS), denoting the presence of both Cu(I) and Cu(II) oxide because of the exposure of the nanoparticles to air [20].

The cells in all the groups were viable, contained markers of apoptosis (bax) and proliferation (PCNA), and secreted substantial amounts of the steroids progesterone, testosterone, and 17*β*-estradiol. The addition of CuNPs/TiO_2_, buckwheat, vitex, rutin, and apigenin was able to alter these parameters.

**Figure 3:**
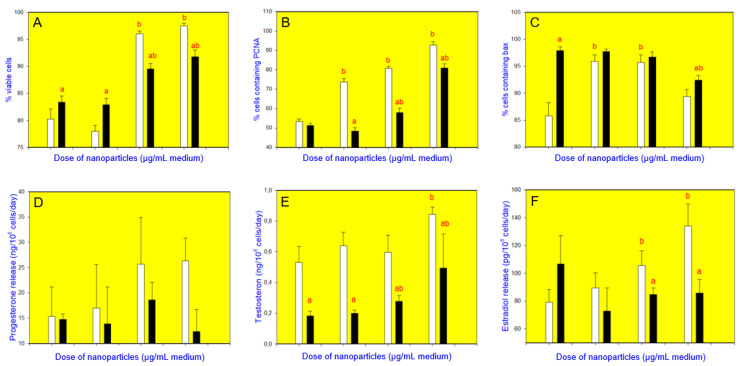
Effect of CuNPs/TiO_2_ (0, 1, 10, or 100 µg/mL) alone (white bars) and in combination with buckwheat (*Fagopyrum esculentum*, 10 µg/mL; black bars) on the viability (Trypan blue exclusion assays) (**A**), proliferation (expression of proliferating cell nuclear antigen, PCNA, quantitative immunocytochemistry) (**B**), apoptosis (expression of bax, quantitative immunocytochemistry) (**C**), and secretion of progesterone (enzyme-linked immunosorbent assay, ELISA) (**D**), testosterone (ELISA) (**E**), and 17*β*-estradiol (ELISA) (**F**) in cultured porcine ovarian granulosa cells. The values are expressed as the mean ± SEM; a: effect of buckwheat–significant (*p* < 0.05) differences between the corresponding groups of cells, treated and untreated with buckwheat and b: effect of CuNPs/TiO_2_–significant (*p* < 0.05) differences between the cells, treated and untreated with CuNPs/TiO_2_.

**Figure 4:**
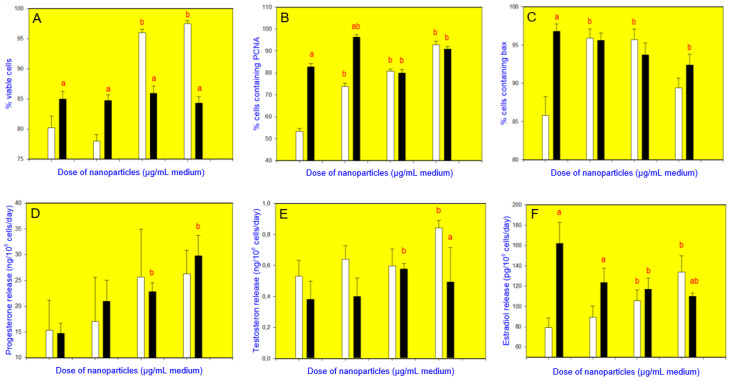
Effect of CuNPs/TiO_2_ (0, 1, 10, or 100 µg/mL) alone (white bars) and in combination with vitex (*Vitex agnus-castus*, 10 µg/mL; black bars) on the viability (Trypan blue exclusion assays) (**A**), proliferation (expression of proliferating cell nuclear antigen, PCNA, quantitative immunocytochemistry) (**B**), apoptosis (expression of bax, quantitative immunocytochemistry) (**C**), and secretion of progesterone (enzyme-linked immunosorbent assay, ELISA) (**D**), testosterone (ELISA) (**E**), and 17*β*-estradiol (ELISA) (**F**) in cultured porcine ovarian granulosa cells. The values are expressed as the mean ± SEM; a: effect of vitex–significant (*p* < 0.05) differences between the corresponding groups of cells, treated and untreated with vitex and b: effect of CuNPs/TiO_2_–significant (*p* < 0.05) differences between the cells, treated and untreated with CuNPs/TiO_2_.

**Figure 5:**
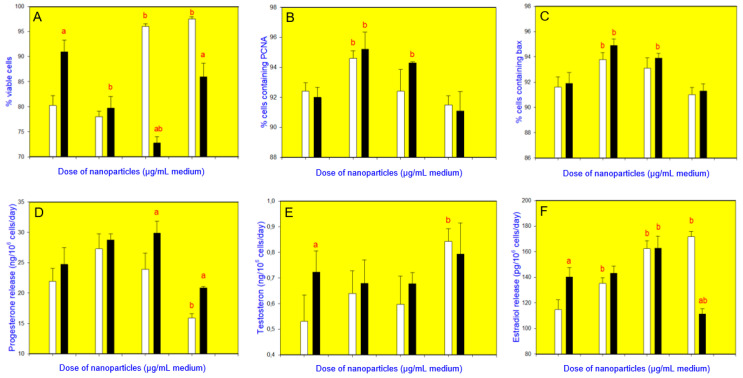
Effect of CuNPs/TiO_2_ (0, 1, 10, or 100 µg/mL) alone (white bars) and in combination with rutin (10 µg/mL; black bars) on the viability (Trypan blue exclusion assays) (**A**), proliferation (expression of proliferating cell nuclear antigen, PCNA, quantitative immunocytochemistry) (**B**), apoptosis (expression of bax, quantitative immunocytochemistry) (**C**), and secretion of progesterone (enzyme-linked immunosorbent assay, ELISA) (**D**), testosterone (ELISA) (**E**), and 17*β*-estradiol (ELISA) (**F**) in cultured porcine ovarian granulosa cells. The values are expressed as the mean ± SEM; a: effect of rutin–significant (*p* < 0.05) differences between the corresponding groups of cells, treated and untreated with rutin and b: effect of CuNPs/TiO_2_–significant (*p* < 0.05) differences between the cells, treated and untreated with CuNPs/TiO_2_.

**Figure 6:**
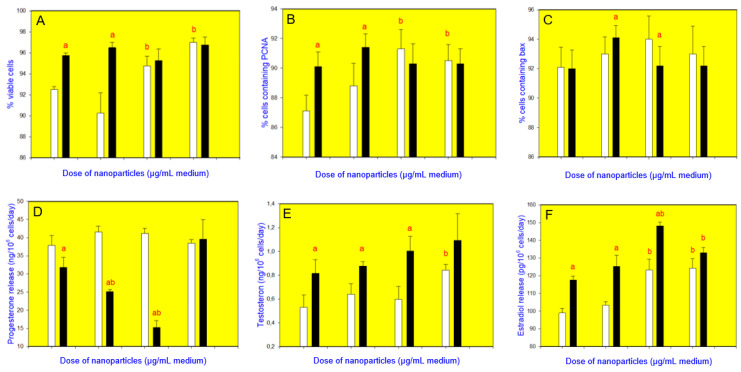
Effect of CuNPs/TiO_2_ (0, 1, 10, or 100 µg/mL) alone (white bars) and in combination with apigenin (10 µg/mL; black bars) on the viability (Trypan blue exclusion assays) (**A**), proliferation (expression of proliferating cell nuclear antigen, PCNA, quantitative immunocytochemistry) (**B**), apoptosis (expression of bax, quantitative immunocytochemistry) (**C**), and secretion of progesterone (enzyme-linked immunosorbent assay, ELISA) (**D**), testosterone (ELISA) (**E**), and 17*β*-estradiol (ELISA) (**F**) in cultured porcine ovarian granulosa cells. The values are expressed as the mean ± SEM; a: effect of apigenin–significant (*p* < 0.05) differences between the corresponding groups of cells, treated and untreated with apigenin and b: effect of CuNPs/TiO_2_–significant (*p* < 0.05) differences between the cells, treated and untreated with CuNPs/TiO_2_.

CuNPs/TiO_2_, when added alone at the doses of 10 or 100 µg/mL, increased cell viability (Figure 3A, Figure 4A, Figure 5A and Figure 6A). It also promoted PCNA accumulation (at all the doses added (Figure 3B and Figure 4B) or at a dose of 1 µg/mL (Figure 5B) or 10 and 100 µg/mL (Figure 6B)). Accumulation of bax was augmented after the addition of CuNPs/TiO_2_ (at 10 and 100 µg/mL, Figure 3C, Figure 4C and Figure 5C), though in one series of experiments CuNPs/TiO_2_ did not affect this marker of apoptosis (Figure 6C). CuNPs/TiO_2_ did not affect progesterone release (Figure 3D, Figure 4D and Figure 6D) except in one series of experiments, where CuNPs/TiO_2_ inhibited it (at the dose of 100 µg/mL, Figure 5D). The testosterone output rose after the addition of CuNPs/TiO_2_ (at the dose of 100 µg/mL, Figure 3E, Figure 4E, Figure 5E and Figure 6E). 17*β*-Estradiol release was promoted by CuNPs/TiO_2_ (at 10 and 100 µg/mL, Figure 3F, Figure 4F, Figure 5F and Figure 6F).

Buckwheat, when added alone, enhanced cell viability (Figure 3A, see CuNPs/TiO_2_ at the dose of 0 µg/mL) and accumulation of bax (Figure 3C), whereas it lowered the testosterone release (Figure 3E). No significant buckwheat influence on PCNA (Figure 3B), progesterone (Figure 3D), and 17*β*-estradiol (Figure 3F) was observed. The influence of CuNPs/TiO_2_ on cell viability (Figure 3A), PCNA/proliferation (Figure 3B), bax/apoptosis (Figure 3C), the release of testosterone (Figure 3E), and 17*β*-estradiol (Figure 3F) was significantly lower in the presence of buckwheat than in its absence. Moreover, buckwheat not only prevented but also even inverted the action of CuNPs/TiO_2_ on bax–CuNPs/TiO_2_ (at 100 µg/mL). The presence of buckwheat did not promote but inhibited bax accumulation (Figure 3C). The presence of buckwheat did not substantially modify the CuNPs/TiO_2_ (1 or 10 µg/mL) effect on the progesterone release, but CuNPs/TiO_2_ added at a dose of 100 µg/mL failed to promote progesterone output in the presence of buckwheat (Figure 3D).

Vitex, when given alone, increased cell viability (Figure 4A), PCNA (Figure 4B) and bax (Figure 4C) accumulation, and the release of 17*β*-estradiol (Figure 4F) but not of progesterone (Figure 4D) or testosterone (Figure 4E). Furthermore, vitex prevented the stimulatory action of CuNPs/TiO_2_ on cell viability (Figure 4A), the accumulation of PCNA (Figure 4B) and bax (Figure 4C), and on the testosterone release (Figure 4E). Moreover, it changed the stimulatory action of CuNPs/TiO_2_ on bax (Figure 4C) and 17*β*-estradiol (Figure 4F) to an inhibitory one. Vitex did not modify the CuNPs/TiO_2_ influence on the progesterone output (Figure 4D).

Rutin addition intensified cell viability (Figure 5A), testosterone (Figure 5E), and 17*β*-estradiol (Figure 5E) release, but not PCNA (Figure 5B) or bax (Figure 5C) accumulation or the release of progesterone (Figure 5D). Furthermore, it arrested and even inverted the CuNPs/TiO_2_ activity on cell viability (Figure 5A), progesterone (Figure 5C), testosterone (Figure 5E), or 17*β*-estradiol (Figure 5F), but not on PCNA (Figure 5B) or bax (Figure 5C) accumulation.

Apigenin promoted cell viability (Figure 6A), PCNA accumulation (Figure 6B), and the secretion of testosterone (Figure 6E) and 17*β*-estradiol (Figure 6F), whereas it shortened the progesterone release (Figure 6D) and had no effect on the accumulation of bax (Figure 6C). In the presence of apigenin, CuNPs/TiO_2_ lost its ability to promote cell viability (Figure 6A), accumulation of PCNA (Figure 6B) and the release of testosterone (Figure 6E), but not to boost the accumulation of bax (Figure 6C) or the release of 17*β*-estradiol (Figure 6F). Moreover, CuNPs/TiO_2_ could inhibit the progesterone release in the presence of apigenin (Figure 6D).

## 4. Discussion

The formation of a monolayer, the high cell viability, the presence of intracellular markers of proliferation and apoptosis and the production of steroid hormones denote that the porcine granulosa cells tested were in good condition and adequate for the analyses and testing of the effects of CuNPs/TiO_2_, plant extracts and phytochemicals. The character of the changes induced by these additives and their combinations are summarized in Table 1. Although the percentage of changes varied between the experiments, all the experiments demonstrated a similar pattern of an additive effect on ovarian cells.

The performed studies demonstrated the stimulatory influence of CuNPs/TiO_2_ on five out of the six measured ovarian cell functions; only one parameter remained unchanged. These observations suggest the absence of toxicity of copper nanoparticles supported on titania, in contrast to a variety of other copper nanoparticles [11,12,14]. Moreover, these results are in line with our previous observation about the stimulatory activity of this type of nanoparticles on ovarian cell functions. The mechanism of this effect and the functional interrelationships between the measured parameters require further elucidation. Nevertheless, CuNPs/TiO_2_ induced an increase of both, proliferation and cytoplasmic apoptosis, suggesting the ability of these nanoparticles to promote ovarian cell turnover. In addition to this, CuNPs/TiO_2_ also increased cell viability, a result that could be rationalized by the dominance of cell proliferation over cell apoptosis. Intensified apoptosis could be a result of the augmented release of testosterone, a promoter of ovarian follicular atresia and cytoplasmic apoptosis. Finally, the growth in proliferation might be due to a larger production of 17*β*-estradiol, which is a known promoter of cell proliferation and viability [56]. In any case, it must be taken into account that cell viability can be affected not only by cytoplasmic apoptosis (induced by bax [53,54]) but, alternatively, by nuclear apoptosis associated with nuclear DNA fragmentation [63,64], which has not been analyzed herein. It has been demonstrated that some regulators can induce the opposite changes in cytoplasmic and nuclear apoptosis in porcine granulosa cells [57]. Therefore, it cannot be completely excluded that CuNPs/TiO_2_ might suppress nuclear apoptosis, which, together with increased proliferation, could boost cell viability. If the stimulatory action of CuNPs/TiO_2_ on the ovary occurred in vivo too, the potential application of CuNPs/TiO_2_ as a safe replacement of toxic CuNPs and a novel biostimulating agent of reproductive processes in animals and humans cannot be disregarded.

The performed studies demonstrate the direct influence of some plant extracts and their phytochemicals on ovarian cell functions. The ability of buckwheat to enhance viability and apoptosis of cultured cells is the first evidence on the direct action of buckwheat on ovarian functions. The mechanisms of these buckwheat effects remain unknown. The stimulatory action of vitex on ovarian cell viability, proliferation, apoptosis, and 17*β*-estradiol release is concordant with previous indications on its ability to increase ovarian cell apoptosis [41,42] and steroidogenesis [36,37,38,39,40]. Furthermore, there is the first evidence on the capability of vitex to promote ovarian cell proliferation, resulting in cell turnover and viability. These effects explain the stimulatory and therapeutic action of vitex on some ovarian functions reported previously [36,37,38,39,40].

The tested phytochemicals exhibit stimulatory activity on some ovarian cell functions similar to the stimulatory effects shown by the whole plant extracts. For example, in our experiments, rutin boosted cell viability and the release of testosterone and 17*β*-estradiol. It cannot be ruled out that the cell viability increase could be attributed to the increased release of the anti-atretic hormone 17*β*-estradiol [62], though the mechanism of rutin’s role is worthy of further studies. The present results are the first evidence about the direct stimulatory activity of rutin on healthy ovarian cells. This activity is in agreement with the therapeutic effect of this polyphenol on an ovary that suffered from ischemia-reperfusion injury [44], restraint [45], and polycystic ovarian syndrome [47], reported previously.

Another polyphenolic phytochemical, apigenin, also manifested stimulatory behavior on several ovarian functions (viability, proliferation, testosterone, and 17*β*-estradiol release), albeit it suppressed the progesterone output. The promotion of cell viability by apigenin can be explained by its capacity to promote ovarian cell proliferation, but not apoptosis, i.e., to change the proliferation/apoptosis rate. This effect could be attributed to the increased secretion of 17*β*-estradiol, which is considered an inducer of ovarian cell proliferation. What is more, the increase of cell viability, proliferation and release of 17*β*-estradiol, and the decrease of progesterone release suggest that apigenin could be a natural stimulating agent of ovarian follicle survival, growth, and an inhibitor of follicular luteinization and atresia, which are under control of these hormones [56]. This is the first proof of the apigenin activity on healthy ovarian cells, which supports the potential benefits of apigenin-containing products for the promotion of animal and human reproductive processes. Furthermore, the changes induced by apigenin in healthy ovarian cells, observed in the present experiments, explain its ability to promote ovarian functions during polycystic ovarian syndrome [51] and ischemia-reperfusion injury [52]. Moreover, the ability of apigenin to induce apoptosis and to reduce the viability of cancer cells, observed previously [50], but not those in healthy ovarian cells (in our experiments), sustains a potential applicability of apigenin for the selective suppression of cancer and for the support of healthy cells in the ovary.

The similar activity found for some plant extracts and plant components on some ovarian cell parameters might indicate, in principle, that the plants tested could affect some ovarian functions because of the presence of the phytochemicals rutin and apigenin among their components. However, only a small proportion of their effects was found to be similar, probably because of the participation of other molecules or molecule complexes. The plants effect could not be explained by the presence of another ubiquitous polyphenolic phytochemical, quercetin, because its suppressive action on all the ovarian functions reported previously [58,59,60,61] is opposed to that observed for buckwheat and vitex in the present study. Therefore, it seems that rutin, apigenin, and quercetin cannot explain the action of buckwheat or vitex on ovarian cells, and that these polyphenols cannot replace the treatment with the whole plant extract.

Interestingly, the plants and the polyphenolic flavonoids tested were able not only to affect basic ovarian cell functions, but also to diminish the stimulatory action of CuNPs/TiO_2_ on these functions. In particular, buckwheat prevented the action of CuNPs/TiO_2_ on all measured indexes and vitex inhibited the activity of CuNPs/TiO_2_ in five out of the six measured parameters, whereas rutin and apigenin did the same in four out of six parameters. None of the plant additives promoted any CuNPs/TiO_2_ effect, which might indicate that the tested plant additives could oppose the action of CuNPs/TiO_2_. Based on our experience on CuNPs [23,24,25,26], we know that their catalytic activity in chemical reactions can be depleted or even completely blocked by their interaction with hydroxyl groups present in organic molecules. Taking into account that plant extracts, as well as rutin and apigenin, are rich in hydroxyl groups, because of the presence of carbohydrate and/or phenolic units, their binding to the surface of the CuNPs could passivate it, with the concomitant reduction of CuNPs’ bioactivity. This behavior could have some health consequences and should be taken into account in view of some potential medical applications, avoiding their joint prescription. Moreover, it cannot be discarded that a daily diet containing these plants and/or their components could reduce the application efficiency of CuNPs/TiO_2_.

The performed experiments did not address all the possible questions concerning the mechanisms of action and interrelationships of the different ovarian regulators. Nevertheless, they show (1) that CuNPs/TiO_2_ could directly stimulate ovarian cell functions; (2) that CuNPs/TiO_2_ could positively affect ovarian cell proliferation, apoptosis, turnover, viability, and steroid hormones release; (3) that the plants buckwheat and vitex, and the phytochemicals rutin and apigenin, could promote some of these ovarian functions too; and (4) that these plant additives could not promote but abate the action of CuNPs/TiO_2_, what must be taken into account for their potential application.

Therefore, given the ability of food and medicinal plants and their polyphenolic constituents to suppress the activity of CuNPs, to intake CuNPs and these plants or plant components jointly seems unadvisable concerning the ovarian functions. However, this interaction can be beneficial to prevent the potential noxious effect, when accidentally exposed to CuNPs, on other types of cells or organs.

## Figures and Tables

**Table 1:** Character of the effect of CuNPs/TiO_2_, buckwheat, vitex, rutin, and apigenin, given alone, on cultured porcine ovarian granulosa cell functions, and the ability of buckwheat, vitex, rutin, and apigenin to modify the activity of CuNPs/TiO_2_^a^.

Additive ^b^	Cell Viability	Proliferation (PCNA)	Apoptosis (Bax)	Release of Steroid Hormones		
				Progesterone	Testosterone	Estradiol
CuNPs/TiO_2_	+	+	+	0	+	+
Buckwheat	+	0	+	0	–	0
Buckwheat and CuNPs/TiO_2_	–	–	–	–	–	–
Vitex	+	+	+	0	0	+
Vitex and CuNPs/TiO_2_	–	–	–	0	–	–
Rutin	+	0	0	0	+	+
Rutin and CuNPs/TiO_2_	–	0	0	–	–	–
Apigenin	+	+	0	–	+	+
Apigenin and CuNPs/TiO_2_	–	–	0	–	–	0

^a^ Type of effect: stimulation (+), no effect (0), and inhibition (–). ^b^ Concentration of CuNPs/TiO_2_: 1, 10, or 100 µg/mL and concentration of buckwheat, vitex, rutin, and apigenin: 10 µg/mL.

## References

[1] Kumar C.S.S.R. (2009). Metallic Nanomaterials.

[2] Reddy L.H., Arias J.L., Nicolas J., Couvreur P. (2012). Magnetic nanoparticles: Design and characterization, toxicity and biocompatibility, pharmaceutical and biomedical applications. Chem. Rev..

[3] Stark W.J., Stoessel P.R., Wohlleben W., Hafner A. (2015). Industrial applications of nanoparticles. Chem. Soc. Rev..

[4] Bhagyaraj S.M., Oluwafemi O.S., Kalarikkal N., Thomas S. (2018). Applications of Nanomaterials: Advances and Key Technologies.

[5] Gawande M.B., Goswami A., Felpin F.X., Asefa T., Huang X., Silva R., Zou X., Zboril R., Varma R.S. (2016). Cu and Cu-based nanoparticles: Synthesis and applications in catalysis. Chem. Rev..

[6] Din M.I., Rehan R. (2017). Synthesis, characterization, and applications of copper nanoparticles. Anal. Lett..

[7] Rafique M., Shaikh A.J., Rasheed R., Tahir M.B., Bakhat H.F., Rafique M.S., Rabbani F. (2017). A review on synthesis, characterization and applications of copper nanoparticles using green method. Nano.

[8] Zhou M., Tian M., Li C. (2016). Copper-based nanomaterials for cancer imaging and therapy. Bioconjugate Chem..

[9] Rathore K., Sharma K. (2018). Biological synthesis of copper nanoparticles and their antimicrobial properties: A review. World J. Pharm. Res..

[10] Verma N., Kumar N. (2019). Synthesis and biomedical applications of copper oxide nanoparticles: An expanding horizon. ACS Biomater. Sci. Eng..

[11] Hejazy M., Koohy M.K., Pour A.B.M., Najafi D. (2018). Toxicity of manufactured copper nanoparticles–a review. Nanomed. Res. J..

[12] Pohanka M. (2019). Copper and copper nanoparticles toxicity and their impact on basic functions in the body. Bratisl. Lek. Listy.

[13] Ameh T., Sayes C.M. (2019). The potential exposure and hazards of copper nanoparticles: A review. Environ. Toxicol. Pharmacol..

[14] Hou J., Wang X., Hayat T., Wang X. (2017). Ecotoxicological effects and mechanism of CuO nanoparticles to individual organisms. Environ. Pollut..

[15] Roychoudhury S., Nath S., Massanyi P., Stawarz R., Kacaniova M., Kolesarova A. (2016). Copper-induced changes in reproductive functions: In vivo and in vitro effects. Physiol. Res..

[16] Yang J., Hu S., Rao M., Hu L., Lei H., Wu Y., Wang Y., Ke D., Xia W., Zhu C. (2017). Copper nanoparticle-induced ovarian injury, follicular atresia, apoptosis, and gene expression alterations in female rats. Int. J. Nanomed..

[17] Zhang C.H., Wang Y., Sun Q.Q., Xia L.L., Hu J.J., Cheng K., Wang X., Fu X.X., Gu H. (2018). Copper nanoparticles show obvious in vitro and in vivo reproductive toxicity via erk mediated signaling pathway in female mice. Int. J. Biol. Sci..

[18] Fevold H.L., Hisaw F.L., Greep R. (1936). Augmentation of the gonad-stimulating action of pituitary extracts by inorganic substances, particularly copper salts. Am. J. Physiol..

[19] Roychoudhury S., Bulla J., Sirotkin A.V., Kolesarova A. (2014). In vitro changes in porcine ovarian granulosa cells induced by copper. J. Environ. Sci. Health A, Tox. Hazard. Subst. Environ. Eng..

[20] Sirotkin A.V., Radosová M., Tarko A., Martín-García I., Alonso F. (2020). Effect of morphology and support of copper nanoparticles on basic ovarian granulosa cell functions. Nanotoxicology.

[21] Alonso F., Yus M. (2008). New synthetic methodologies based on active transition metals. Pure Appl. Chem..

[22] Deka P., Borah B.J., Saikia H., Bharali P. (2019). Cu-based nanoparticles as emerging environmental catalysts. Chem. Rec..

[23] Abdulkin P., Moglie Y., Knappett B.R., Jefferson D.A., Yus M., Alonso F., Wheatley A.E.H. (2013). New routes to Cu(I)/Cu nanocatalysts for the multicomponent click synthesis of 1,2,3-triazoles. Nanoscale.

[24] Alonso F., Arroyo A., Martín-García I., Moglie Y. (2015). Cross-dehydrogenative coupling of tertiary amines and terminal alkynes catalyzed by copper nanoparticles on zeolite. Adv. Synth. Catal..

[25] Mitrofanov A.Y., Murashkina A.V., Martín-García I., Alonso F., Beletskaya I.P. (2017). Formation of C-C, C-S and C-N bonds catalysed by supported copper nanoparticles. Catal. Sci. Technol..

[26] Martín-García I., Alonso F. (2018). Synthesis of dihydroindoloisoquinolines through the copper-catalyzed cross-dehydrogenative coupling of tetrahydroisoquinolines and nitroalkanes. Chem. Eur. J..

[27] Kenneth L., Thanh P.H., Doan D.L. (2017). Interaction of plant extracts with central nervous system receptors. Medicines.

[28] Dhama K., Karthik K., Khandia R., Munjal A., Tiwari R., Rana R., Khurana S.K., Ullah S., Khan R.U., Alagawany M. (2018). Medicinal and therapeutic potential of herbs and plant metabolites/extracts countering viral pathogens—Current knowledge and future prospects. Curr. Drug Metab..

[29] Pohl F., Lin P.K.T. (2018). The potential use of plant natural products and plant extracts with antioxidant properties for the prevention/treatment of neurodegenerative diseases: In vitro, in vivo and clinical trials. Molecules.

[30] Quero J., Marmol I., Rodriguez-Yoldi M.J., Cerrada E. (2020). Insight into the potential application of polyphenol-rich dietary intervention in degenerative disease management. Food Funct..

[31] Akour A., Kasabri V., Afifi F.U., Bulatova N. (2016). The use of medicinal herbs in gynecological and pregnancy-related disorders by Jordanian women: A review of folkloric practice vs. evidence-based pharmacology. Pharm. Biol..

[32] Sabourian R., Karimpour-Razkenari E., Saeedi M., Bagheri M.S., Khanavi M., Sadati N., Akbarzadeh T., Ardekani M.R. (2016). Medicinal plants used in iranian traditional medicine (itm) as contraceptive agents. Curr. Pharm. Biotechnol..

[33] Bruno L.O., Simoes R.S., de Jesus Simoes M., Girão M.J.B.C., Grundmann O. (2018). Pregnancy and herbal medicines: An unnecessary risk for women’s health-A narrative review. Phytother Res..

[34] Kam P.C., Barnett D.W., Douglas I.D. (2019). Herbal medicines and pregnancy: A narrative review and anaesthetic considerations. Anaesth. Intensive Care..

[35] Giménez-Bastida J.A., Zielinski H., Piskula M., Zielinska D., Szawara-Nowak D. (2017). Buckwheat bioactive compounds, their derived phenolic metabolites and their health benefits. Mol. Nutr. Food Res..

[36] Ahangarpour A., Najimi S.A., Farbood Y. (2016). Effects of Vitex agnus-castus fruit on sex hormones and antioxidant indices in a D-galactose-induced aging female mouse model. J. Chin. Med. Assoc..

[37] Kakadia N., Patel P., Deshpande S., Shah G. (2018). Effect of Vitex negundo L. seeds in letrozole induced polycystic ovarian syndrome. J. Tradit. Complement. Med..

[38] Moini Jazani A., Nasimi Doost Azgomi H., Nasimi Doost Azgomi A., Nasimi Doost Azgomi R. (2019). A comprehensive review of clinical studies with herbal medicine on polycystic ovary syndrome (PCOS). Daru.

[39] Rafieian-Kopaei M., Movahedi M. (2017). Systematic review of premenstrual, postmenstrual and infertility disorders of vitex agnus castus. Electron. Physician..

[40] Csupor D., Lantos T., Hegyi P., Benkő R., Viola R., Gyöngyi Z., Csécsei P., Tóth B., Vasas A., Márta K. (2019). Vitex agnus-castus in premenstrual syndrome: A meta-analysis of double-blind randomised controlled trials. Complement. Ther. Med..

[41] Ohyama K., Akaike T., Hirobe C., Yamakawa T. (2003). Cytotoxicity and apoptotic inducibility of Vitex agnus-castus fruit extract in cultured human normal and cancer cells and effect on growth. Biol. Pharm. Bull..

[42] Zhou Y., Liu Y.E., Cao J., Zeng G., Shen C., Li Y., Zhou M., Chen Y., Pu W., Potters L. (2009). Vitexins, nature-derived lignan compounds, induce apoptosis and suppress tumor growth. Clin. Cancer Res..

[43] Abdallah H.M., El-Halawany A.M. (2018). Rutin isolated from chrozophora tinctoria enhances bone cell proliferation and ossification markers. Oxid. Med. Cell. Longev..

[44] Wei S.M., Yan Z.Z., Zhou J. (2011). Protective effect of rutin on testicular ischemia-reperfusion injury. J. Pediatric Surg..

[45] Mehfooz A., Wei Q., Zheng K., Fadlalla M.B., Maltasic G., Shi F. (2018). Protective roles of Rutin against restraint stress on spermatogenesis in testes of adult mice. Tissue Cell.

[46] Aksu E.H., Kandemir F.M., Özkaraca M., Ömür A.D., Küçükler S., Çomaklı S. (2017). Rutin ameliorates cisplatin-induced reproductive damage via suppression of oxidative stress and apoptosis in adult male rats. Andrologia.

[47] Hu T., Yuan X., Ye R., Zhou H., Lin J., Zhang C., Zhang H., Wei G., Dong M., Huang Y. (2017). Brown adipose tissue activation by rutin ameliorates polycystic ovary syndrome in rat. J. Nutr. Biochem..

[48] Yan X., Qi M., Li P., Zhan Y., Shao H. (2017). Apigenin in cancer therapy: Anti-cancer effects and mechanisms of action. Cell Biosci..

[49] Ozbey U., Attar R., Romero M.A., Alhewairini S.S., Afshar B., Sabitaliyevich U.Y., Hanna-Wakim L., Ozcelik B., Farooqi A.A. (2018). Apigenin as an effective anticancer natural product: Spotlight on TRAIL, WNT/β-catenin, JAK-STAT pathways, and microRNAs. J. Cell. Biochem..

[50] Tavsan Z., Kayali H.A. (2019). Flavonoids showed anticancer effects on the ovarian cancer cells: Involvement of reactive oxygen species, apoptosis, cell cycle and invasion. Biomed. Pharmacother..

[51] Darabi P., Khazali H., Mehrabani Natanzi M. (2019). Therapeutic potentials of the natural plant flavonoid apigenin in polycystic ovary syndrome in rat model: Via modulation of pro-inflammatory cytokines and antioxidant activity. Gynecol. Endocrinol..

[52] Soyman Z., Kelekçi S., Sal V., Şevket O., Bayındır N., Uzun H. (2017). Effects of apigenin on experimental ischemia/reperfusion injury in the rat ovary. Balkan Med. J..

[53] Peña-Blanco A., García-Sáez A.J. (2018). Bax, Bak and beyond – mitochondrial performance in apoptosis. FEBS J..

[54] Sharma A., Boise L.H., Shanmugam M. (2019). Cancer metabolism and the evasion of apoptotic cell death. Cancers.

[55] Wang S.C. (2014). PCNA: A silent housekeeper or a potential therapeutic target?. Trends Pharmacol. Sci..

[56] Sirotkin A.V. (2014). Regulators of Ovarian Functions.

[57] Pavlová S., Klucska K., Vašíček D., Kotwica J., Sirotkin A.V. (2011). Transcription factor NF-κB (p50/p50, p65/p65) controls porcine ovarian cells functions. Anim. Reprod. Sci..

[58] Sirotkin A.V., Hrabovszká S., Štochmaľová A., Grossmann R., Alwasel S., Halim Harrath A. (2019). Effect of quercetin on ovarian cells of pigs and cattle. Anim. Reprod. Sci..

[59] Sirotkin A.V., Štochmaľová A., Alexa R., Kádasi A., Bauer M., Grossmann R., Alrezaki A., Alwasel S., Harrath A.H. (2019). Quercetin directly inhibits basal ovarian cell functions and their response to the stimulatory action of FSH. Eur. J. Pharmacol..

[60] Tarko A., Štochmalova A., Hrabovszka S., Vachanova A., Harrath A.H., Alwasel S., Grossman R., Sirotkin A.V. (2018). Can xylene and quercetin directly affect basic ovarian cell functions?. Res. Vet. Sci..

[61] Tarko A., Štochmaľová A., Jedličková K., Hrabovszká S., Vachanová A., Harrath A.H., Alwasel S., Alrezaki A., Kotwica J., Baláži A. (2019). Effects of benzene, quercetin, and their combination on porcine ovarian cell proliferation, apoptosis, and hormone release. Arch. Anim. Breed..

[62] Strober W. (2001). Trypan blue exclusion test of cell viability. Curr. Protoc. Immunol..

[63] Cissen M., van Wely M.V., Scholten I., Mansell S., de Bruin J.P., Mol B.W., Braat D., Repping S., Hamer G. (2016). measuring sperm DNA fragmentation and clinical outcomes of medically assisted reproduction: A systematic review and meta-analysis. PLoS ONE.

[64] Lee J.M., Park J.H., Kim B.Y., Kim I.-H. (2018). Terminal deoxynucleotidyl transferase-mediated deoxyuridine triphosphate nick end labeling (TUNEL) assay to characterize histopathologic changes following thermal injury. Ann. Dermatol..

